# Supplementary material for: Intrathecal catheter implantation decreases cerebrospinal fluid dynamics in cynomolgus monkeys
Source: PLoS One. 2020 Dec 30;15(12):e0244090. doi: 10.1371/journal.pone.0244090 (PMC7773283; doi:10.1371/journal.pone.0244090)
Supplement: S1 File — (DOCX) [file pone.0244090.s002.docx]

**Competing interests:**

I have read the journal's policy and the authors of this manuscript have the following competing interests: This study was funded in part by Voyager Therapeutics. Authors BAM and GRS are employed by Alcyone Therapeutics. GRS was employed by Voyager Therapeutics during the course of this study. JRZ is a fulltime employee of Northern Biomedical. BAM has received grant support from Voyager Therapeutics, Genentech, Alcyone Lifesciences, Biogen, and Minnetronix; BAM is a member of the Neurapheresis Research Consortium. BAM is scientific advisory board member for Alcyone Lifesciences, Chiari and Syringomyelia Foundation, The International Society for Hydrocephalus and CSF Disorders, The International CSF Dynamics Society, and has served as a consultant to Voyager Therapeutics, Praxis Medicines, Roche, SwanBio Therapeutics, CereVasc, Minnetronix, Invicro, Genentech, Medtrad Biosystems, Behavior Imaging, Neurosyntek, and Cerebral Therapeutics. There are no patents, products in development or marketed products to declare. This does not alter our adherence to PLOS ONE policies on sharing data and materials.

This work was supported by Voyager Therapeutics, National Institutes of Health, National Institute of General Medical Sciences grant P20GM103408 and 4U54GM104944-04 and the University of Idaho, Vandal Ideas Project. Publication of this article was funded by the University of Idaho Open Access Publishing Fund. The funders had no role in study data collection and analysis, decision to publish, or preparation of the manuscript. Voyager Therapeutics had a role in the study design. Authors BAM and GRS are employed by Alcyone Therapeutics. GRS was employed by Voyager Therapeutics during the course of this study. Alcyone Therapeutics provided support in the form of salary for authors BAM and GRS, but did not have any additional role in the study design, data collection and analysis, decision to publish, or preparation of the manuscript. Voyager Therapeutics provided support in the form of salary for author GRS. Author JRZ is employed by Northern Biomedical Research. Northern Biomedical Research provided support in the form of salary for author JRZ, but did not have any additional role in the study design, data collection and analysis, decision to publish, or preparation of the manuscript. The specific roles of these authors are articulated in the ‘author contributions’ section
